# Deformation Mechanisms of Magnesium Alloys with Rare-Earth and Zinc Additions under Plane Strain Compression

**DOI:** 10.3390/ma17010033

**Published:** 2023-12-21

**Authors:** Xun Zeng, Sangbong Yi

**Affiliations:** 1 Department of Mechanical Engineering, University of Southampton, Southampton SO17 1BJ, UK; xun.zeng@soton.ac.uk; 2 Institute of Materials and Process Design, Helmholtz-Zentrum Hereon, 21502 Geesthacht, Germany

**Keywords:** dislocation slip, twinning, magnesium alloys, texture

## Abstract

The introduction of rare-earth (RE) elements into magnesium (Mg) alloys can significantly improve their ductility, thereby extending the applications of Mg products. However, the impacts of their chemical composition, temperature and processing methods on the mechanical properties of Mg products are highly debatable. In this work, we systematically investigate the deformation behaviors of Mg–Nd and Mg–Zn–Nd alloys using electron backscattered diffraction (EBSD) characterization. The samples were deformed to different stress levels to study the microstructure and texture development during channel die compression. The results reveal that the room temperature formability of the Mg–Nd alloy can be enhanced with the addition of Zn. This is attributed to the higher activities of prismatic slip and tensile twinning in the Mg–Zn–Nd alloy as compared to the binary counterpart, facilitating strain accommodation. When the strain increases, the growing and merging of the same twin variant rapidly consumes the parent grain, which is responsible for the texture modification from the transverse to the basal direction. At elevated temperatures, the twinning is suppressed in both alloys due to the decreased critical resolved shear stress of the non-basal slip systems. Additionally, an obvious sigmoidal yielding phenomenon is observed due to the multiple activation of the different deformation modes. These findings offer valuable insights into the evolution of the microstructure and texture during plane strain compression, elucidating the connections between material chemical composition, processing and mechanical properties, which are important for the advancement of Mg alloy application.

## 1. Introduction

Due to compelling needs for weight saving and environmentally friendly materials, magnesium (Mg) and its alloys, known as lightest-structural materials, have drawn significant attention, especially in the automobile and aerospace industries. However, their limited formability, which stems from their hexagonal close-packed (HCP) lattice structure, presents challenges that researchers and industries must address. Compared to the face-centered cubic (FCC) and body-centered cubic (BCC) crystal structures, HCP crystals have much fewer slip systems for accommodating plastic strain, which leads to premature cracking. The formability limitation in Mg alloys is particularly pronounced at room temperature (RT). This is because basal slip and tensile twinning, as the primary deformation modes due to their relatively lower critical resolved shear stress (CRSS) than at other temperatures, are insufficient to accommodate the strain [1,2].

To harness the benefits of magnesium’s low density while overcoming its formability challenges, numerous studies have been carried out regarding material design and thermomechanical treatments. Among these approaches, rare-earth (RE) additions have been proven to be an effective way of enhancing the formability of Mg alloys. RE additions can influence both recrystallization and deformation behaviors. The effects of RE on recrystallization are usually attributed to the weakened recrystallization texture, which reduces the anisotropy of Mg alloys and facilitates strain accommodation. These texture-weakening effects of RE arise from the recrystallized nuclei that originated in particles [3,4], twins [5] and shear bands [6,7], which show different orientations to the deformed grains, known as oriented nucleation theory. Additionally, preferential grain growth theory suggests that some specific oriented recrystallized grains have a growth advantage over others [8]. In terms of deformation, the introduction of yttrium (Y) to Mg alloys was reported to reduce the basal stacking fault energy [9,10], promoting non-basal (prismatic and pyramidal) slip. Li et al. [11] proposed that Ce amplified the role of pyramidal <a> slip over prismatic slip during plane strain compression, leading to the formation of RE texture. Moreover, RE addition can affect the CRSS value of the dislocation slip and twinning systems [12], facilitating the activation of various deformation modes. It was reported that Y elements in solid solutions significantly increase the CRSS of basal and tensile twinning in Mg alloys because solute atoms hinder atomic shuffling, while the CRSS of prismatic slip remains stable due to the reduced stacking fault energy [13]. This, in turn, reduces the stress/strain accumulation that initiates cracking. In terms of the effects of RE on the precipitation strengthening, it was reported that intermetallics can refine the grain size of the α-Mg matrix [14,15], resulting in the change of fracture mode from cleavage to ductile. Furthermore, the segregation [16,17] and precipitation [18] of RE atoms reduce the grain boundary mobility, changing the dynamic and static recrystallization textures.

Understanding the effects of RE addition on the mechanical response of Mg alloys is crucial for the development of high-performance Mg alloys. However, no consensus has yet been reached because of the variations in chemical composition and the experimental conditions. For instance, the influence of RE can be magnified or eliminated by different third alloying elements, and the deformation mechanisms can differ between room temperature and elevated temperatures. To address these challenges, two Mg–RE alloys were used in this study. Neodymium (Nd), the chosen RE element, is known for its excellent texture-weakening effect [19], which enhances the ductility of Mg alloys. The third alloying element, zinc (Zn), which is one of the most common alloying elements, introduces long-range periodic stacking fault ordering (LPSO) phases [20] by reducing the stacking fault energy. In contrast to the conventional uniaxial tension or compression tests, channel die compression (CDC), characterized by plane strain deformation wherein one of the material flows is restricted, is applied. This method, with its similarity to rolling in terms of strain path, can stimulate the microstructure and texture developments, shedding light on the deformation mechanisms that are present during rolling. This research addresses the knowledge gaps in the interplay of chemical composition, mechanical properties, dislocation slip and twinning activities during plane strain compression. It holds significant practical importance, as it closely simulates the rolling process and provides valuable insights into the production of Magnesium sheets.

## 2. Materials and Methods

**Figure 1:**
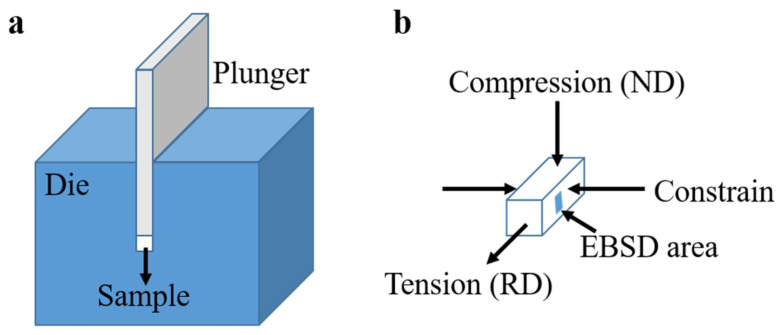
Schematic of channel die compression. (**a**) Experimental setup, (**b**) sample alignment.

The examined materials were cut from two rolled Mg sheets with chemical compositions of Mg-1.7Nd (wt.%) and Mg-1.1Zn-1.7Nd (wt.%), as shown in Table 1. They are termed N2 and ZN12, respectively. After recrystallization annealing at 450 °C for 1 h, the rectangular samples were machined with a diameter of 5 (ND) × 5 (TD) × 6 (RD) mm^3^; ND, TD and RD refer to the normal direction, transverse direction and rolling direction of the rolled sheets. Figure 1a demonstrates the setup of CDC, which can be installed inside a furnace for elevated temperature tests. The samples were aligned to achieve plain strain condition (compression along ND and tension along RD), as shown in Figure 1b. The CDC tests were carried out using a universal testing machine (Zwick Z050, ZwickRoell, Ulm, Germany) equipped with a heating furnace. The strain rate was kept at 0.001 s^−1^ for all samples while the room temperature (RT) and elevated temperature of 200 °C (ET) were employed to study the effects of temperature on the mechanical responses of two alloys. To investigate the microstructure and texture evolution during CDC, a stress-controlled deformation strategy was introduced. The deformation was manually interrupted at three distinct stress levels after yielding (at 15 MPa beyond the yield strength), at the ultimate strength and at an intermediate stress (mean value of yield strength and ultimate strength). For example, the loading of the N2 RT ultimate strength sample was terminated at a predefined upper force limit of 11,700 N, given the sample cross-sectional area of 30 mm^2^ and US of 390 MPa. The intention was to explore the dominant deformation mechanisms in the different deformation stages of CDC.

The samples were ground and polished on the RD-ND plane. Prior to EBSD characterization, the samples were electrolytically polished using a commercially available AC2 electrolyte and Lectropol-5 machine (Struers, Champigny sur Marne cedex, France) at −20 °C and 30 V. The EBSD characterizations were performed using a field emission scanning electron microscope (Zeiss Crossbeam 550L, ZEISS Group, Oberkochen, Germany) with a step size of 0.4 µm.

## 3. Results

### 3.1. Grain Information of the Initial Condition

**Figure 2:**
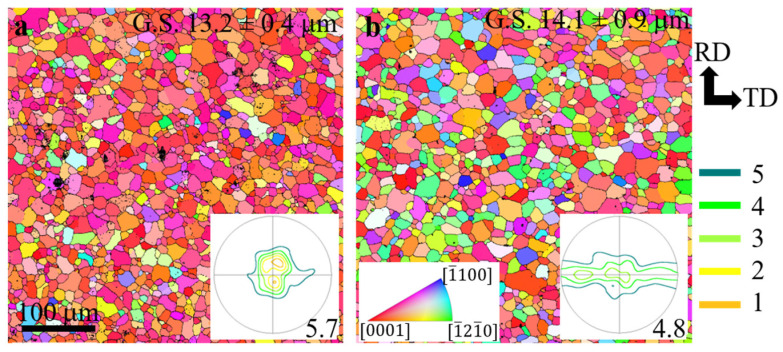
EBSD grain and texture maps of the alloys prior to deformation: (**a**) N2, and (**b**) ZN12.

Figure 2 shows the microstructure and texture of the N2 and ZN12 alloys prior to CDC. It is clear that both samples are fully recrystallized without any twins. There is no obvious difference in the average grain size between the N2 (Figure 2a) and ZN12 (Figure 2b) samples. Therefore, the effect of the grain size on deformation behaviors can be excluded. However, most of the grains in the N2 sample appear in red, indicating that their c-axes are aligned parallel to ND. This alignment is further confirmed by the pole figure, which exhibits a weak basal texture with two basal peaks that tilt slightly from ND to RD. For the sake of brevity, the same alignment of the sample coordinate with RD and TD parallel to the vertical and horizontal directions was applied to all EBSD maps afterwards. In contrast, the ZN12 sample contains many green and blue grains, and the corresponding texture consists of a basal component and a wide TD-spread component. The texture intensities are 5.7 for the N2 and 4.8 for the ZN12 alloys, which are much lower as compared to the strong basal texture seen in commercial Mg alloys [21]. This suggests that Nd weakens the recrystallization texture of Mg alloys, and this texture-weakening effect is further strengthened with the Zn addition.

### 3.2. Mechanical Properties of Channel Die Compression Samples

**Figure 3:**
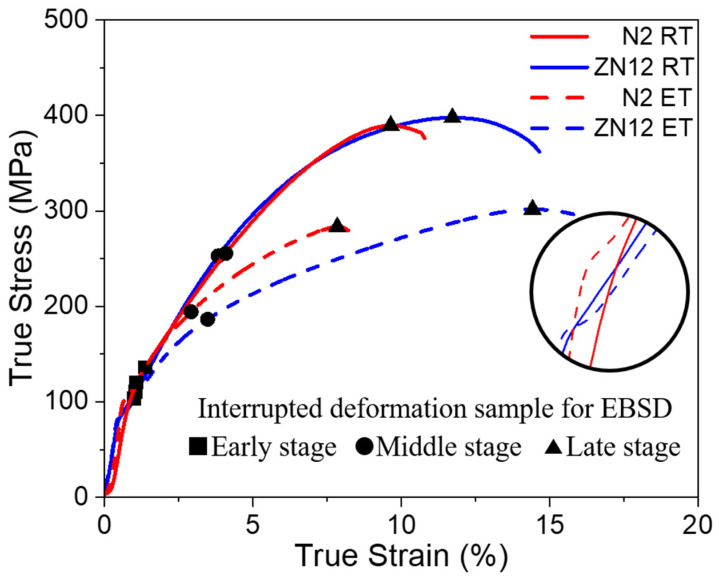
Engineering stress-strain curves of the N2 and ZN12 alloys deformed at RT and ET. The magnified view of the curves near yielding strength are superimposed. The interrupted deformation samples for the EBSD measurements are marked with different symbols on the stress-strain curves.

The stress-strain curves and detailed mechanical property data for the CDC samples are shown in Figure 3 and Table 2. The ZN12 samples exhibit a larger fracture strain than the N2 sample at both RT and ET, indicating a positive effect of Zn addition on the formability of Mg-RE alloy. Additionally, the US of two alloys are quite comparable, while the YS of the ZN12 is lower than its counterpart. This difference is attributed, to some extent, to the favorable TD orientation of the ZN12 sample, resulting in a high resolved-shear stress for basal slip and tensile twinning. Moreover, the heat treatment reduced the YS and US of two alloys as compared to those of the RT samples due to the enhanced mobility of the dislocations at the elevated temperature. An obvious sigmoidal-yielding phenomenon, as superimposed in Figure 3, is observed in the ET samples, while the elastic-plastic deformation transition is more smooth in the RT samples. The CDC processes were interrupted for EBSD measurements in order to investigate the microstructure and texture evolutions of the early, middle and late deformation stages, as marked with rectangle, circle and triangle symbols, respectively.

### 3.3. Deformation Behaviors of the Room Temperature Samples

**Figure 4:**
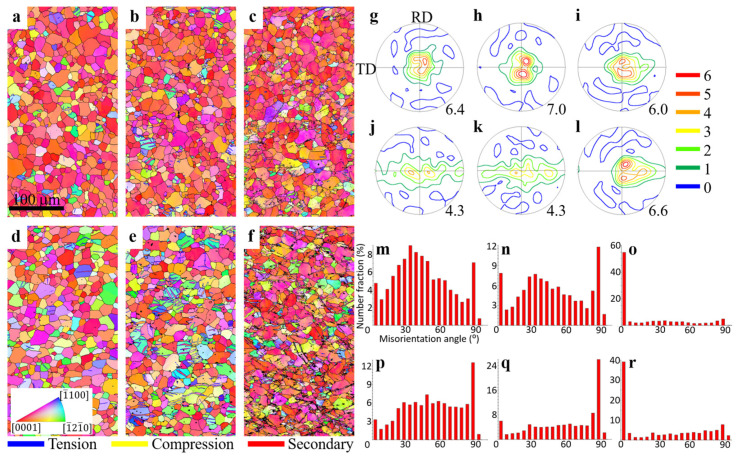
EBSD maps of the room temperature channel die compression samples in different deformation stages. Grains maps of the (**a**–**c**) N2 and the (**d**–**f**) ZN12 sample in the early, middle and late deformation stages. (**g**–**l**) Corresponding texture maps of the N2 and ZN12 samples. (**m**–**r**) Corresponding misorientation angle distribution.

The EBSD maps of the RT samples in different deformation stages are presented in Figure 4. In the early stages, the microstructures and textures of the N2 (Figure 4a,g) and ZN12 (Figure 4d,j) are very similar to the fully recrystallized condition (Figure 2) due to the relatively low stress/strain level (15 MPa over yield strength). With further loading to the middle deformation stages, many lamellar-shaped structures (marked with blue grain boundaries) are observed. These lamellar structures are indexed as tension twins as they exhibit a misorientation of 86° about <$11\bar{2}0$> axis to their neighbors, which is also confirmed by the high peaks in the grain boundary distribution maps (Figure 4n,q). The other two common twin types in Mg alloys, compression and secondary twins, are separately highlighted with yellow and red boundaries. Obviously, tension twins dominate in both samples. It is worth noting that the twinning activities are more pronounced in the ZN12 as compared to the N2 sample. As indicated in Table 3, the middle deformation stage of the ZN12 RT sample (Figure 4e) exhibits 85 twinned grains, constituting a number fraction of 20.6%. This fraction is nearly double that which is observed in the N2 sample (Figure 4b). Despite the high number of twins, their contribution to the texture is limited due to their small twinned volume (Figure 4h,k). In the late deformation stage of the N2 sample (Figure 4c), more tension twins are formed as well as some tiny compression twins (yellow boundaries) and secondary twins (red boundaries) are observed. This is because the stress concentration in the late deformation stages exceeds the CRSS of different twinning modes, stimulating twin nucleation. However, the boundary length fraction of tension twins in the ZN12 decreases from 19% (Figure 4e) to 1.2% (Figure 4f) with increasing stress. This may be attributed to the high dislocation density at the twin boundaries, which acts as a barrier to dislocation movements. It results in a poor indexing rate of twin boundaries, as indicated by the black bands in Figure 4f. Another possible explanation is the fast growth of the twins. The entire parent grains are consumed by the twins so that the twin boundaries disappear when they reach the grain surface. The basal texture of the N2 sample is maintained in the late deformation stages (Figure 4i). In the ZN12 sample, two basal pole peaks tilted into RD are formed while the TD component is significantly weakened (Figure 4l). This texture modification is attributed to the reorientation of tension twins by 86°. Due to the high dislocation density at the US points, numerous low-angle grain boundaries derived from dislocation pile-ups play a preliminary role in the misorientation angle plots (Figure 4o,r). The ZN12 exhibits a higher number fraction of tension twin boundaries than its counterpart.

### 3.4. Deformation Behaviors of the Elevated Temperature Samples

**Figure 5:**
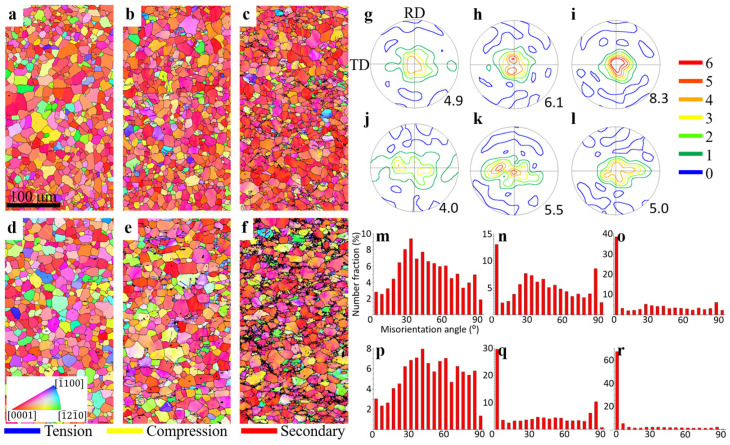
EBSD maps of the elevated temperature channel die compression samples in different deformation stages. Grains maps of the (**a**–**c**) N2 and (**d**–**f**) ZN12 samples in the early, middle and late deformation stages. (**g**–**l**) Corresponding texture maps of the N2 and ZN12 samples. (**m**–**r**) Corresponding misorientation angle distribution.

Figure 5 demonstrates the EBSD maps of the ET samples in different deformation stages. As is the case for the RT samples, there are no significant differences between the early stage of the ET samples and the fully recrystallized condition. As opposed to the profuse twins in the RT samples (Figure 4b,e), however, twinning activities are less-developed in the middle deformation stages of the ET samples (Figure 5b,e). This phenomenon can be related to the decreased CRSS of the non-basal slip at the elevated temperature [22], where strain is primarily accommodated by basal and non-basal slip at ET. This aligns with the observed large orientation gradient, as indicated by the color change in the grains, because dislocation slip can generally reorient the grain. The orientation gradient is particularly obvious in the ZN12 sample, with the TD-oriented grains showing a mixture of green and yellow regions. As a result, the TD spread texture component becomes more concentrated towards the ND (Figure 5k). Due to the high stress and strain levels of the ZN12 sample in late deformation stages, a considerable area fraction of non-indexed area is observed (Figure 5f). Compared to the RD split texture at RT (Figure 4l), a weak basal texture is found in the ZN12 sample (Figure 5l).

## 4. Discussion

### 4.1. Effects of Dislocation Slip on the Deformation Process

The experimental results reveal differences in the deformation behaviors between binary and ternary Mg–Nd alloys under CDC. Understanding the underlying mechanisms of these varying mechanical response in the two alloys is crucial for developing high-performance Mg alloys. The ZN12 alloy with a favorable TD spread texture facilitates the activation of basal slip and tensile twinning in comparison to the N2 sample, contributing to the observed lower yielding strength. As the strain increases, the basal components of both alloys are strengthened. However, the N2 sample predominantly undergoes deformation through basal slip, causing the reorientation of the basal plane parallel to the rolling direction. In contrast, the texture development in the ZN12 sample is achieved through the growth of tension twins. The advanced nucleation and growth of twins in ZN12 help to release the local strain accumulation, resulting in improved ductility as compared to N2. At the elevated temperature, atoms are more thermally activated, enabling them to overcome the bonding force and enhancing the dislocation mobility. Consequently, the YS decreases with increasing temperature. In addition, it has been reported that the cross slip from basal to non-basal planes is promoted at ET [23,24]. This is confirmed by the sigmoidal shapes of the stress-strain curve in Figure 3, which indicate the activation of various deformation modes. The dislocation tangles resulting from the interaction of different deformation modes will hinder their mobility, leading to the increased slop of stress-strain curve (sigmoidal yielding phenomenon). It is expected that the formability of the two alloys will be improved at the elevated temperature. However, a higher fracture strain is only observed in the ZN12 sample. One possible explanation for this is that the enhanced non-basal slip in the N2 sample cannot fully compensate for the reduced contribution of twins at elevated temperatures. In contrast, the ZN12 sample retains a considerable number of twins, which ensures better formability.

**Figure 6:**
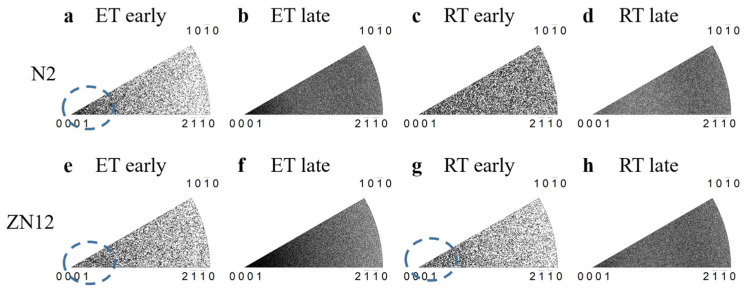
IGMA distributions of the CDC samples at different temperature and deformation stages. The circles in the figures indicate the <0001> peak of prismatic slip.

The next consideration is regarding which deformation modes are activated. It is generally accepted that basal slip, which the lowest CRSS in all deformation modes, serves as the primary deformation mode regardless of the deformation temperature. To investigate the active dislocation modes, an intragranular misorientation analysis (IGMA) approach is introduced. This method is based on the theory of lattice rotation by slip system. That is, when slip system is activated, it will induce a lattice rotation around a specific crystallographic axis [25,26], known as the Taylor axis. The dominant slip mode can be determined by matching the Taylor axis to the measured IGMA distribution. The prismatic slip in the HCP structure shares the same <0001> Taylor axis in all three variants, while the basal and pyramidal slips show various rotation axes with different slip variants. Thus, a high concentration of IGMA at the <0001> axis indicates the high activity of prismatic slip. Figure 6 exhibits the IGMA maps of the CDC samples with a misorientation angle range of 1 to 3°. The threshold is set to exclude noise inherent in traditional EBSD characterization with a precision of 0.5° and the effects of the low-angle grain boundaries. Figure 6 exhibits the IGMA maps of the CDC samples. In the early stages of CDC at ET, both the N2 (Figure 6a) and ZN12 (Figure 6e) samples show an IGMA peak at the <0001> axis, which means that prismatic slip is highly activated immediately after yielding. This is in contrast to previous studies which suggested that basal slip and tension twinning are the dominant deformation modes of Mg alloys at RT, while prismatic slip can hardly operate in the plane strain compression of even Mg alloys with favorable orientations [27,28]. However, our results reveal that the RE addition significantly promotes the activity of prismatic slip in both the N2 and ZN12 ET samples. This preference for prismatic slip persists throughout the entire deformation process, as intense peaks are observed in the late deformation stages (Figure 6b,f). However, only the ZN12 sample has a concentration at RT (Figure 6g), while the IGMA map of the N2 RT sample is homogeneously distributed (Figure 6c). This indicates that the third alloying element Zn magnified the effects of Nd on the activity of prismatic slip. Because prismatic slip is highly activated in the ZN12 sample, it reduces the contribution of basal slip to strain accommodation. This is beneficial for the ductility of the ZN12, as basal dislocations are prone to accumulate at grain boundaries and lead to premature cracking.

### 4.2. Effects of Twinning on the Deformation Process

**Figure 7:**
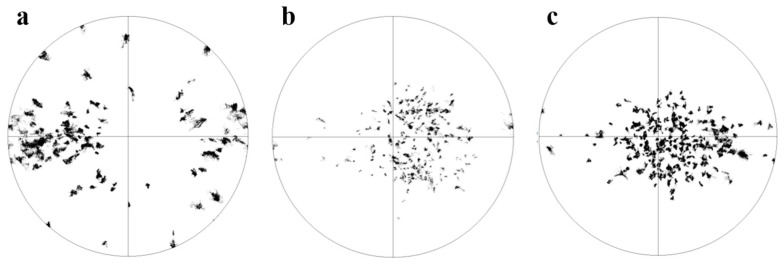
Orientations of the RT ZN12 sample in the middle deformation stage: (**a**) parent grains, (**b**) twins and (**c**) twin-free grains.

Twinning plays a pivotal role in the deformation of the Mg alloys, facilitating strain accommodation along the c-axis, while the dominant deformation mode of basal slip is confined to the basal planes. First, a twin network can separate the parent grain into several subgrains, introducing grain refinement strengthening. Twin nucleation and growth can also help to release local stress and strain. Finally, twins introduce new orientations that facilitate the activation of deformation modes. Under elevated temperatures, a notable reduction in the CRSS of non-basal slip systems occurs, promoting the activation of non-basal slip mechanisms. Consequently, the relative contributions of twinning to deformation process diminishes at elevated temperatures as twinning is less sensitive to temperature change. This comprehensive elucidation offers a thorough understanding of the observed suppression of twinning in Mg alloys at higher temperatures. Because twinning is a polar deformation mode which only occurs along twin directions, the orientations of parent grains have significant effects on twinning activities. Profuse twins are observed in the middle deformation stage of the ZN12 RT sample (Figure 4e). Due to the moderate stress level in middle deformation stage, it is reasonable to assume that parents are larger than twins in the twinned grains. The orientations of the parent grains, twins and twin-free grains are manually extracted and shown in Figure 7. Obviously, most of the parents are TD-oriented, whereas the basal poles of the twins are slightly tilted from ND to RD (RD split texture). This observation aligns with the fact that TD grains undergo compression load along the a-axis, corresponding to the tension along the c-axis, which is favorable for tension twinning. In addition, Jeong [29] reported that the pile-up of prismatic dislocation can dissociate into basal dislocation and stacking fault, promoting the nucleation of tensile twinning. Thus, a more advanced twinning activity is observed in the ZN2 with higher prismatic slip and more favorable orientation than that observed in the N2 sample. As a result, the parent grains are reoriented to form the RD split texture. In contrast, the twin-free grains are compressed along ND, suppressing the tension twinning.

**Figure 8:**
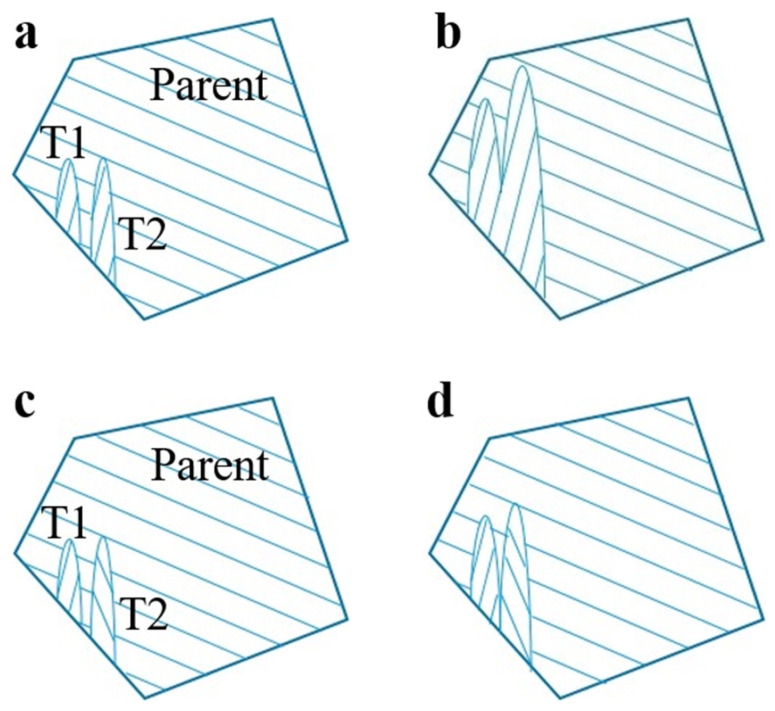
Growth of tension twin variants: (**a**,**b**) twin variants with an equivalent rotation axis. (**c**,**d**) twin variants with different rotation axis. The lines in the grain indicate basal planes.

With increasing stress, it is expected that more twins will be formed in accompany with the growth of the present twins. However, our result shows a decrease in the total twin count of the ZN12 RT sample from the middle to late deformation stages (Figure 4e,f). Meanwhile, the texture intensities significantly increase from 4.3 to 6.6 MRD (multiple of random distribution). This may be related to the rapid growth of the twins. In Figure 4e, there are 85 twinned grains with 130 tension twins. Among these, 41 of the twinned grains show a single twin variant, which allows them to grow freely by consuming the parent grain. However, the other twinned grains exhibit two or more twin variants. Depending on the twin variant type, different twin variants can either grow and merge into a large twin, or they can impede the twin growth of each other. For example, Figure 8a shows that the ($\bar{1}102$) [$1\bar{1}01$] tension twin variant T1 shares the equivalent [$11\bar{2}0$] rotation axis with the ($\bar{1}10\bar{2}$) [$1\bar{1}0\bar{1}$] twin variant T2. Their misorientation angle is only 7.4°, and they can merge into a large twin as deformation progresses (Figure 8b). In another case, T2 is indexed as a ($\bar{1}01\bar{2}$) [$10\bar{1}\bar{1}$] variant, showing a misorientation of 60° to T1 (Figure 8c). With such a high misorientation angle, the twin boundary movement will be hindered (Figure 8d). The combination of single twin variant and equivalent twin variants are observed to dominate the twinning behaviors. As a result, the parent grains are replaced by the twins, leading to the RD split texture (Figure 4e).

To conclude, the results indicate that the incorporation of Zn and Nd into Mg–RE alloys promotes prismatic slip and tensile twinning during plane strain compression, leading to a substantial enhancement in ductility. It has practical implications for Mg sheet production when using rolling process with the same strain state. For example, because twinning decreases the tendency of cracking at RT, we can introduce more twins with a minor pre-strain to fabricate Mg sheets with the same composition. This reduces cost and time as compared to the previous method that require the deformation of the sample at an elevated temperature.

## 5. Conclusions

In this study, the deformation behaviors of the Mg–RE alloys under plane strain compression are investigated. Distinct mechanical responses were observed between the binary Mg–Nd and its ternary counterpart with the Zn addition. To explain the underlying mechanisms, EBSD characterizations were applied to different deformation stages of the two alloys. The main conclusions are drawn as follows:

(1) Compared to the Mg–Nd binary alloy, the addition of Zn can significantly improve the ductility of the sample at room and elevated temperatures. The yield strength of the ZN12 is slightly lower than that of the N2, while their ultimate strengths are comparable.
(2) A high activity of prismatic slip is observed in early deformation stage of the ZN12 sample. The multiple activation of different deformation modes, characterized by the sigmoidal yielding phenomenon, are beneficial to the strain accommodation.
(3) The ZN12 sample shows a more advanced twinning activity than its counterpart, due to its favorable orientation and its dissociation of prismatic slip. However, the rapid growth of the same twin variant consumes the parent grain, leading to the orientation change from TD to basal components.

## Figures and Tables

**Table 1:** Chemical composition (wt.%) of the examined alloys.

Alloy	Nd	Zn	Mg
Mg-Nd (N2)	1.75	-	Balanced
Mg-Zn-Nd (ZN12)	1.71	1.07	Balanced

**Table 2:** Mechanical properties of the CDC samples.

	Yield Strength (MPa)	Ultimate Strength (MPa)	Fracture Strain (%)
N2 RT	120 ± 3.1	390 ± 6.8	11.1 ± 0.3
N2 ET	104 ± 2.2	283 ± 4.0	8.3 ± 0.4
ZN12 RT	95 ± 3.2	398 ± 7.3	14.9 ± 0.4
ZN12 ET	87 ± 1.8	302 ± 6.6	17.7 ± 0.2

**Table 3:** Number fraction of the twinned grains under different deformation stages.

	Early Stage	Middle Stage	Late Stage
N2 RT	4.2	10.5	13.0
N2 ET	0	6.1	5.3
ZN12 RT	6.9	20.6	18.8
ZN12 ET	0.2	13.3	8.7

## Data Availability

Experimental methods and results are available from the authors.
